# Association of Self-Reported Functional Limitations among a National Community-Based Sample of Older United States Adults with Pain: A Cross-Sectional Study

**DOI:** 10.3390/jcm10091836

**Published:** 2021-04-23

**Authors:** David R. Axon, Darlena Le

**Affiliations:** Department of Pharmacy Practice and Science, College of Pharmacy, The University of Arizona, Tucson, AZ 85721, USA; dle@pharmacy.arizona.edu

**Keywords:** older adults, pain, functional limitation

## Abstract

The characteristics of self-reported functional limitations among older United States (US) adults with pain are currently unknown. This cross-sectional study aimed to determine the characteristics associated with functional limitations among non-institutionalized older (≥50 years) US adults with pain using 2017 Medical Expenditure Panel Survey (MEPS) data. Eligible subjects were alive for the calendar year, aged ≥50 years, and experienced pain within the past four weeks. Hierarchical logistic regression models were utilized to determine significant characteristics associated with functional limitations (outcome variable; yes, no). Functional limitations included difficulty with bending, stooping, climbing stairs, grasping objects, lifting, reaching overhead, standing for long periods of time, or walking. Extrapolation of national data values was possible by adjusting for the complex MEPS design. We found approximately 22 million of the 57 million older US adults (≥50 years) who reported pain had a functional limitation in 2017. Characteristics associated with functional limitations included: gender, race, ethnicity, employment status, marital status, pain intensity, physical health, number of chronic conditions, and frequent exercise status. Knowledge of characteristics associated with functional limitations may provide an opportunity to identify and resolve gaps in patient care among this population.

## 1. Introduction

Approximately 126 million United States (US) adults were affected by pain in the past three months in 2012 [1]. Pain management is often burdensome and involves multiple treatment domains (including both pharmacological and non-pharmacological approaches) [2]. Pain is well known to interfere with people’s functional capacity [3,4,5,6]. For example, older adults with musculoskeletal conditions (e.g., osteoarthritis, chronic back pain) have reported issues in specific activities of daily living (ADL) domains such as doing heavy housework, bending or kneeling down, and climbing stairs without walking aids [3]. Restricting back pain has been strongly linked to mobility disability as related to walking a quarter of a mile, climbing stairs, and carrying 10 pounds [4]. Among older adults suffering from pain due to arthritis, there was a greater likelihood of reports of upper-lower extremity functional limitations (e.g., extending arms above shoulder level, pulling/pushing large objects, picking up a coin) and ADL disability (e.g., bathing, eating, walking, using the toilet) [5]. Moreover, the distribution of the pain, from no sites to widespread pain, has been associated with new onset of mobility difficulty [6].

The correlation between pain and functional capacity, as established by prior studies [3,4,5,6], indicates the importance of investigating the factors that are associated with functional limitations in order to develop interventions that minimize the impact of pain on functional capacity later in life. Prior studies have investigated specific medical conditions [3,4,5] and their impact on the functional capacity of older adults. However, there is a lack of research determining the characteristics associated with functional limitations among older US adults with pain. This information may enable healthcare providers and caregivers to better identify unmet needs for older adults with functional limitations.

The objective of this study was to determine the characteristics associated with self-reported functional limitations for a national sample of older (aged ≥50 years) US adults with self-reported pain.

## 2. Methods

### 2.1. Study Design and Study Data

This study employed a cross-sectional design and utilized the latest available Medical Expenditure Panel Survey (MEPS) data from 2017 [7]. MEPS data are collected during multiple rounds of interviews over a two-year period using a subsample of the previous years’ National Health Interview Survey (NHIS). Disabled and minority groups are purposefully oversampled and appropriate weightings can be applied to furnish nationally representative estimates of the non-institutionalized US population. This study used one of the key MEPS components, the MEPS household component (MEPS-HC). MEPS-HC contains data about each household member surveyed, including demographics, employment, income, health insurance coverage, health conditions, and health status, among others [8]. 

### 2.2. Study Eligibility

The full-year consolidated data file for 2017 was used to identify eligible subjects. Eligible subjects were those who were alive for the full calendar year, ≥50 years of age, and reported pain in the last four weeks. Pain was identified by a response to the question “During the past four weeks, pain interfered with normal work outside the home and housework” of: (1) a little bit, (2) moderately, (3) quite a bit, or (4) extremely [9,10].

### 2.3. Independent Variables

Independent variables were classified into one of four groups (outlined below) according to Andersen’s Behavioral Model of Health Services Use. This model was initially developed by Ronald M. Andersen in the 1960s to help define access and use of healthcare services, and to assist in the analysis of national survey data, among other purposes. Since then, the model has been refined and used in many studies. For this study, the model was used to help determine and organize relevant variables to include in the analyses into one of four categories: predisposing factors, enabling factors, need factors, and personal health practices and external environmental factors. Predisposing factors typically include an individual’s demographic characteristics. Enabling factors typically include variables that assist an individual to access the healthcare system. Need factors typically include variables that indicate health services are needed. Personal health practices factors and external environmental factors are more recent additions to the model that can also influence an individual’s use of the healthcare system [11].

In this study, predisposing factors consisted of: gender (female, male); age (≥65 years, 50–64 years); race (White, other); and ethnicity (non-Hispanic, Hispanic).

Enabling factors consisted of: education level (<high school, high school, >high school); employment status (employed, unemployed); health insurance coverage (private, public, uninsured); income (to indicate federal poverty level: poor/near poor/low income (<200% federal poverty level); middle/high income (≥200% federal poverty level)); and marital status (married, other).

Need factors included: perceived pain intensity (little/moderate, quite a bit/extreme); perceived mental health condition (excellent/very good/good, fair/poor); perceived physical health condition (excellent/very good/good, fair/poor); and total number of the following prevalent chronic health conditions: angina, arthritis, asthma, cancer, chronic bronchitis, coronary heart disease, diabetes, joint pain, emphysema, hypercholesterolemia, hypertension, myocardial infarction, other unspecified heart disease, stroke (0, 1, 2, 3, 4, ≥5).

Personal health practices and environmental factors were frequent physical activity status (at least 30 min of moderate to vigorous intensity exercise five times per week; yes, no), current smoking status (yes, no), and US census region (Northeast, Midwest, South, West) [9,10].

### 2.4. Outcome Variable

The outcome variable in this study was self-reported presence (yes or no) of a functional limitation, which was defined as having difficulty with physical actions such as bending or stooping, climbing stairs, grasping objects, lifting, reaching overhead, standing for long periods of time, or walking [9,10].

### 2.5. Data Analysis

Data analysis was conducted to compare subjects with a functional limitation to those without a functional limitation using chi-square tests. Logistic regression models were constructed to assess characteristics that had a statistically significant association with reporting a functional limitation, with no functional limitation serving as a reference group. Using a hierarchical approach, additional groups of factors (enabling, need, personal health practices and environmental) were added to the initial model that contained the predisposing factors until a fully adjusted model was constructed. An alpha level of 0.05 was set a priori. Analyses were conducted using SAS University Edition (SAS institute Inc., Cary, NC, USA).

## 3. Results

**Figure 1:**
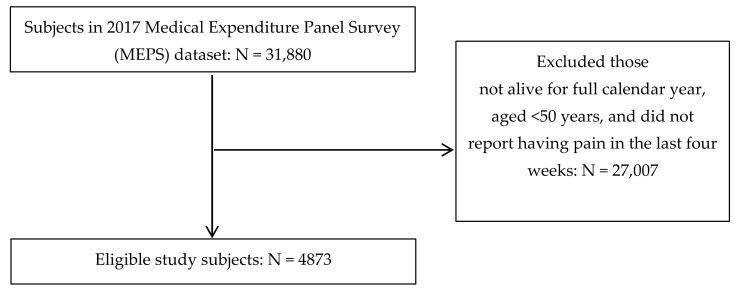
Flowchart of subject eligibility criteria.

This study included 4873 eligible subjects from a total of 31,880 subjects available in the 2017 MEPS dataset (Figure 1). Of these, 2011 reported a functional limitation while 2862 did not. In the weighted population of 57,051,651 people, 39.3% (95% confidence interval (CI) = 37.5%, 41.1%) reported a functional limitation.

As reported in Table 1, the majority of study subjects had the following characteristics: female (55.3%), aged ≥65 years (51.0%), White race (81.1%), non-Hispanic ethnicity (89.9%), greater than high school education level (50.7%), unemployed (61.0%), private health insurance coverage (61.0%), middle/high income (67.8%), married (57.2%), little/moderate perceived pain intensity (75.1%), excellent/very good/good mental health (85.5%), excellent/very good/good perceived physical health (73.0%), ≥4 total prevalent chronic health conditions (50.4%), no frequent physical activity (58.1%), and non-smokers (85.1%). The most common US census region was the south (38.2%). There were differences between those who had a functional limitation and those who did not for all characteristics (*p* < 0.05) except race (*p* = 0.5393).

Table 2 reports the logistic regression results. Among predisposing factors, females were approximately 1.2 times more likely (adjusted odds ratio (AOR) = 1.24, 95% CI = 1.04, 1.47) to report a functional limitation than men, those who were of White race (versus other race) were approximately 1.2 times more likely (AOR = 1.24, 95% CI = 1.03, 1.50) to report a functional limitation, and those who were of non-Hispanic (versus Hispanic) ethnicity were approximately 1.7 times likely to report a functional limitation (AOR = 1.69, 95% CI = 1.31, 2.18). Among enabling factors, those who were employed (versus unemployed) and those who were married (versus other marital status) were less likely to report a functional limitation (AOR = 0.51, 95% CI = 0.41, 0.64 and AOR = 0.57, 95% CI = 0.48, 0.67, respectively). Among need factors, those who reported little/moderate perceived pain intensity (versus quite a bit/extreme pain intensity) and those who reported excellent/very good/good perceived physical health (versus fair/poor physical health) had a lower likelihood of reporting a functional limitation (AOR = 0.36, 95% CI = 0.29, 0.44 and AOR = 0.48, 95% CI = 0.39, 0.59). Likewise, having fewer chronic conditions was associated with a lower likelihood of reporting a functional limitation (ranging from AOR = 0.11 for 0 versus ≥5 conditions to AOR = 0.66 for 4 versus ≥5 conditions). Among personal health practices and environmental factors, those who reported doing frequent physical activity were less likely to report a functional limitation (AOR = 0.74, 95% CI = 0.62, 0.88). The final logistic regression model had a c-statistic of 0.80 and Wald statistic of <0.0001.

## 4. Discussion

This study adds new information to the literature about the prevalence and characteristics associated with functional limitations among a nationally representative sample of older US adults with pain. The key finding in this study was that several factors, in particular need factors, proved to be associated with reporting a limitation.

Among predisposing factors, non-Hispanics had the highest odds of reporting a limitation, which was closely followed by those who were White. In a previous study, it was shown that compared to non-Hispanic Black, Hispanic, and non-Hispanic Asian adults, non-Hispanic White adults were more likely to suffer from pain [12]. In particular, pain that affects multiple musculoskeletal sites among older adults may cause their mobility performance to decline [6]. Of note, a prior study had contrasting results in which there were greater reports among African Americans with pain of experiencing functional limitations compared to other racial/ethnic groups [13]. This result may be due to use of ineffective pain coping strategies among African Americans [14]. Moreover, patient underreporting among minorities may be a contributing factor to pain management inconsistencies, leading to insufficient pain management [15]. Due to the mixed results and other influential factors that may be at play, further investigation of the role of race and ethnicity on functional limitations among older adults with pain may be necessary.

For the predisposing factor of gender, females had a higher likelihood of reporting a functional limitation. Gender differences in functional limitation may be due to certain pain conditions that interfere with women’s physical ability. For instance, among women and men with knee osteoarthritis, women exhibited poorer knee extension [16]. Older women have also been found to have a higher incidence of back pain contributing to restrictive activity [17]. Additionally, with back pain, in a cohort study of older White women, researchers discovered that slow walking speed and slow chair stand time can mediate the association between frequent back pain and all-cause mortality [18]. This study highlights the impact of functional ability on long-term health.

Of the enabling factors in this study, two were associated with a higher likelihood of reporting a limitation: unemployment and other marital status. First, for unemployment, this finding is perhaps not surprising as the study population was comprised of older adults who most likely are unemployed/retired. As age increases among older adults, labor force participation rate decreases since the combination of health conditions and a physically demanding job can make working more challenging [19]. Disability is the most common cause of not being in the workforce among older adults [19]. Types of disabilities that can affect the older population include vision, hearing, cognition, ambulatory, self-care, and independent living [20].

For the second enabling factor, marital status, this study revealed that those who were not married were more likely to report a functional limitation. Since marital status and living arrangements are often linked to one another, for older adults who live alone, these individuals may encounter more challenges if they have difficulty performing daily activities [20]. In a previous study which considered marital status as a component of social isolation, there was an association found between mortality risk and not being married [21]. Social isolation and loneliness have also been associated with decreased gait speed, with loneliness also linked to increased ADL difficulties [22]. Besides the social isolation and loneliness that may affect those who are not married, these individuals also experience higher affective pain [23], a potential explanation to this study’s finding where unmarried older adults with pain were more likely to suffer from a functional limitation.

All need factors, except for perceived mental health condition, were associated with having a functional limitation. Significant need factors included perceived pain intensity, perceived physical health condition, and total number of prevalent chronic health conditions.

For perceived pain intensity, this study found that those with quite a bit/extreme pain were more likely to report a limitation than those with little/moderate pain. This finding aligns with the findings of previous studies. For example, the Indonesian Family Life Survey comprising of adults aged at least 50 years found that participants who reported severe pain had higher functional limitation scores and weaker grip strength [24], while another study found that pain severity was associated with activities of daily living limitations [3]. Notably, for certain painful conditions, such as lumbopelvic pain, higher levels of pain severity negatively impacted walking ability [25].

For perceived physical health condition, this study found that those who reported their physical health condition as “fair” or “poor” compared to those who reported “excellent”, “very good” or “good” were more likely to report a limitation. This finding also aligns well with another study that found that among older adults with a history of falls, poor self-reported health was significantly linked to ADL limitation [26]. Falls have been linked to pain as it has been found that among older adults, there was a high fall risk in those with two or more pain sites [27]. The importance of self-rated health and its influence on health outcomes for certain medical conditions has also been studied. For example, participants in a study were evaluated pre-surgery (for osteoarthritis) and at 3 and 6 months post-surgery [28]. For all time points, worse past self-reported health was associated with worse future physical health [28].

For total number of prevalent chronic health conditions, this study found that having five or more prevalent chronic conditions was associated with a greater likelihood of a functional limitation than having fewer than five prevalent chronic health conditions. The number of prevalent chronic conditions had one of the strongest associations with functional limitations in this study. A previous study analyzing older adults found that the addition of a chronic condition was linked to an increase in the number of functional limitations [29]; for instance, diabetic neuropathy can be classified as painful [30] and is known to be associated with walking difficulties [31]. Furthermore, presence of major chronic diseases among older adults has been correlated with higher rates of incident disability with ADL items, such as bathing, walking, toileting, and eating [32].

It was interesting that perceived mental health condition was not associated with reporting a functional limitation; previous research also using MEPS data found that having any type of limitation was associated with lower likelihood of an individual reporting good mental health (AOR = 0.51, 95% CI = 0.38, 0.68) [33]. Thus, further research into the association between types of limitations and mental health status may be warranted.

Lastly, among the personal health practices and environmental factors, only physical activity status was significantly associated with functional limitation—specifically, older adults with pain and who reported infrequent physical activity had higher odds of reporting a functional limitation. As mentioned earlier, pain is widely recognized in its ability to affect people’s functional capacity [3,4,5,6]. This current study highlights the importance of assessing physical activity status in pain management as doing so will allow identification of older adults at higher risk of experiencing a functional limitation. Physical inactivity has been found to be one of the major risk factors related to disability-adjusted life-years [34]. This finding was consistent with a prior study which found that among middle-aged and older adults, those that completed at least 5 min per day of moderate to vigorous physical activity demonstrated better chair stand time and handgrip strength (for women) compared to those with less than 5 min per day of moderate to vigorous physical activity [35]. Notably, one study also found that among older adults with physical limitations, persistent mobility disability occurred less among those who underwent a physical activity program (goal of 150 min per week) versus those assigned to a health education workshop program [36].

Based on the present study’s findings, there are several potential recommendations for healthcare providers to help optimize pain management. Since several of the need factors discussed earlier were associated with functional limitations among older US adults, addressing these specific areas may aid in targeting pain management strategies that will be most helpful for this population. These strategies may extend beyond prescribed medications to include medical procedures, physical, psychological, and other strategies [2,37]. First, for pain intensity, because greater pain intensity is associated with having a functional limitation, the ability to offer pain treatment as soon as possible may help to promote better patient outcomes in functional performance. Among patients with pain, early intervention has been found to result in lower pain intensity and fewer disability days [38]. Moreover, perceived physical health was also associated with functional limitations and, thus, an indication that it may be helpful for healthcare providers to obtain patient self-reports on their physical health. Further investigation may be needed in this area due to research findings with mixed results. For instance, a prior study found that among older people, both self-reported and objective measures of physical functioning were associated with disability onset [39], whereas another study among people with knee osteoarthritis found physical performance test measures correlated poorly with self-reported measures [40]. For the last significant need factor, total number of prevalent chronic health conditions, since having more chronic conditions was associated with reporting a functional limitation, this suggests the potential benefit for healthcare providers to recommend older adults with five or more chronic conditions to undergo physical performance tests. This strategy would allow for implementation of early therapeutic intervention when indicated. Based on a previous study, patients with chronic lower back pain who received early physical therapy intervention had improved functionality, which included components such as pain intensity, walking, self-care, and lifting [41].

Overall, recommendations such as these may help to enhance the ability of healthcare providers and caregivers to identify vulnerable individuals at greater risk for having a functional limitation, and perhaps prevent the consequences that having a functional limitation can entail. However, future research is required to determine whether there will be differences in pain treatment outcomes if these recommendations and interventions were to be implemented. Findings from future research may help to further improve current clinical practice guidelines for the treatment of pain. Future work should also focus on how to support older adults with pain and functional limitations, to move this field forward.

This study had some limitations. One inherent limitation was that this study was a cross-sectional study that utilized existing MEPS data. Thus, cause-and-effect relationships cannot be concluded from this study. Recall bias may also occur since MEPS utilizes data reported by participants, and participants’ ability to understand questions and provide an appropriate response may vary across individuals. Self-reported data may also vary from measured or observed data. Although the study included several variables, there may be others that were not available in the dataset that could also be associated with functional limitations, for example, gait speed. The definitions of pain and limitations in this study were broad, thus it was not possible to distinguish between individuals with different types of pain (e.g., acute versus chronic pain), or the types of limitations, which may have influenced the results. Likewise, the definition for several variables such as exercise was dichotomous, which may limit their value. For other dichotomous variables such as race and ethnicity, this may leave the impression that it is the skin color or language spoken that is associated with a greater risk of reporting a functional limitation. Data were analyzed using a hierarchical logistic regression approach, yet other more flexible approaches such as the change-in-estimate method could have produced difference results. Finally, the findings of this study are generalizable only to non-institutionalized US adults aged at least 50 years with pain, so cannot be generalized to younger age groups or those outside the US.

## 5. Conclusions

To conclude, given the scarcity of existing research, this study provides new information about the factors that were associated with the presence of limitations among a nationally representative sample of community-dwelling older (aged ≥50 years) US adults with pain. Several factors were found to be associated with functional limitations in this study, with the strongest being the total number of prevalent chronic health conditions. Future work to investigate reasons why some of these factors are associated with limitations in this population is warranted.

## Figures and Tables

**Table 1:** Characteristics of older United States adults (age ≥50 years) with self-reported pain in the past four weeks, stratified by presence of a self-reported functional limitation versus no functional limitation.

Variables			Total Weighted N = 57,051,651		*p*
			Functional Limitation (Weighted N = 22,417,233) Weighted % (95% CI)	No Functional Limitation (Weighted N = 34,634,419) Weighted % (95% CI)	
Predisposing factors:					
	Gender				<0.0001
		Female	60.4 (57.8–62.9)	51.9 (50.2–53.7)	
		Male	39.6 (37.1–42.2)	48.1 (46.3–49.8)	
	Age				<0.0001
		≥65 years	59.8 (57.1–62.6)	45.3 (42.9–47.7)	
		50–64 years	40.2 (37.4–42.9)	54.7 (52.3–57.1)	
	Race				0.5393
		White	80.7 (78.7–82.7)	81.4 (79.4–83.4)	
		Other	19.3 (17.3–21.3)	18.6 (16.6–20.6)	
	Ethnicity				<0.0001
		Non-Hispanic	92.4 (91.0–93.9)	88.3 (86.6–90.0)	
		Hispanic	7.6 (6.1–9.0)	11.7 (10.0–13.4)	
Enabling factors:					
	Education level				<0.0001
		<High school	19.6 (17.4–21.8)	14.1 (12.6–15.6)	
		High school	33.6 (31.4–35.7)	32.7 (30.6–34.7)	
		>High school	46.8 (44.1–49.6)	53.2 (50.8–55.7)	
	Employment status				<0.0001
		Employed	20.3 (17.7–22.9)	51.1 (48.5–53.7)	
		Unemployed	79.7 (77.1–82.3)	48.9 (46.3–51.5)	
	Health insurance coverage				<0.0001
		Private	50.7 (47.9–53.6)	67.7 (65.6–69.8)	
		Public	47.0 (44.2–49.8)	27.9 (25.9–29.9)	
		Uninsured	2.3 (1.6–3.0)	4.4 (3.6–5.2)	
	Income				<0.0001
		Poor/near poor/low income	42.5 (39.4–45.7)	25.5 (23.5–27.5)	
		Middle/high income	57.5 (54.3–60.6)	74.5 (72.5–76.5)	
	Marital status				<0.0001
		Married	46.0 (43.0–49.0)	64.4 (62.2–66.5)	
		Other	54.0 (51.0–57.0)	35.6 (33.5–37.8)	
Need factors:					
	Perceived pain intensity				<0.0001
		Little/moderate	56.5 (53.6–59.4)	87.1 (85.5–88.6)	
		Quite a bit/extreme	43.5 (40.6–46.4)	12.9 (11.4–14.5)	
	Perceived mental health condition				<0.0001
		Excellent/very good/good	77.5 (75.4–79.7)	90.7 (89.6–91.8)	
		Fair/poor	22.5 (20.3–24.6)	9.3 (8.3–10.4)	
	Perceived physical health condition				<0.0001
		Excellent/very good/good	56.5 (54.0–59.1)	83.6 (82.1–85.2)	
		Fair/poor	43.5 (40.9–46.0)	16.4 (14.8–17.9)	
	Total number of prevalent chronic health conditions				<0.0001
		0	0.8 (0.4–1.3)	7.5 (6.2–8.8)	
		1	3.3 (2.2–4.3)	14.6 (13.2–16.0)	
		2	11.3 (9.4–13.1)	18.5 (16.7–20.2)	
		3	14.7 (13.0–16.5)	21.6 (19.8–23.3)	
		4	17.8 (15.8–19.8)	15.7 (14.3–17.2)	
		≥5	52.1 (49.3–54.9)	22.2 (20.4–23.9)	
Personal health practices and environmental factors:					
	Frequent physical activity status				<0.0001
		Yes	31.6 (29.0–34.2)	48.6 (46.2–51.0)	
		No	68.4 (65.8–71.0)	51.4 (49.0–53.8)	
	Current smoking status				0.0098
		Yes	16.7 (14.9–18.4)	13.7 (12.2–15.2)	
		No	83.3 (81.6–85.1)	86.3 (84.8–87.8)	
	US census region				0.0458
		Northeast	17.0 (14.6–19.4)	18.9 (16.9–21.0)	
		Midwest	22.7 (20.3–25.2)	21.6 (19.4–23.9)	
		South	40.7 (37.8–43.6)	36.6 (33.9–39.3)	
		West	19.5 (17.2–21.9)	22.8 (20.4–25.3)	

Analyses are based on 4873 (unweighted) older United States adults (age ≥50 years) alive during the calendar year 2017 with self-reported pain in the past four weeks. Differences between the functional limitation group (unweighted *n* = 2011) and no limitation group (unweighted *n* = 2862) based on chi-square tests. Abbreviations: % = percentage; CI = confidence interval.

**Table 2:** Characteristics associated with functional limitations among older United States adults (age ≥50 years) with self-reported pain in the past four weeks.

Factors			Adjusted Odds Ratio (95% Confidence Interval)
Predisposing factors:			
	Gender		
		Female	**1.24 (1.04, 1.47)**
		Male	**Reference**
	Age		
		≥65 years	1.01 (0.84, 1.22)
		50–64 years	Reference
	Race		
		White	**1.24 (1.03, 1.50)**
		Other	**Reference**
	Ethnicity		
		Non-Hispanic	**1.69 (1.31, 2.18)**
		Hispanic	**Reference**
Enabling factors:			
	Education level		
		<High school	0.84 (0.67, 1.05)
		High school	0.89 (0.75, 1.06)
		>High school	Reference
	Employment status		
		Employed	**0.51 (0.41, 0.64)**
		Unemployed	**Reference**
	Health insurance coverage		
		Private	1.23 (0.79, 1.92)
		Public	1.46 (0.94, 2.26)
		Uninsured	Reference
	Income		
		Poor/near poor/low income	0.99 (0.82, 1.21)
		Middle/high income	Reference
	Marital status		
		Married	**0.57 (0.48, 0.67)**
		Other	**Reference**
Need factors:			
	Perceived pain intensity		
		Little/moderate	**0.36 (0.29, 0.44)**
		Quite a bit/extreme	**Reference**
	Perceived mental health condition		
		Excellent/very good/good	0.89 (0.70, 1.13)
		Fair/poor	Reference
	Perceived physical health condition		
		Excellent/very good/good	**0.48 (0.39, 0.59)**
		Fair/poor	**Reference**
	Total number of prevalent chronic health conditions		
		0	**0.11 (0.06, 0.20)**
		1	**0.20 (0.13, 0.30)**
		2	**0.43 (0.33, 0.56)**
		3	**0.45 (0.36, 0.56)**
		4	**0.66 (0.53, 0.84)**
		≥5	**Reference**
Personal health practices and environmental factors:			
	Frequent physical activity status		
		Yes	**0.74 (0.62, 0.88)**
		No	**Reference**
	Current smoking status		
		Yes	0.95 (0.77, 1.17)
		No	Reference
	US census region		
		Midwest	0.94 (0.70, 1.27)
		Northeast	1.16 (0.87, 1.54)
		South	1.07 (0.83, 1.37)
		West	Reference

Analyses are based on 4873 (unweighted) older United States adults (age ≥50 years) alive during the calendar year 2017 with self-reported pain in the past four weeks. The reference group for the dependent variable in the binomial logistic regression was “no functional limitation” (N = 2862). The model had a Wald statistic of *p* < 0.0001 and a c-statistic of 0.80. Bold indicates a statistically significant association with functional limitations.

## Data Availability

Data are publicly available on the MEPS website.
